# Structure of the S100A4/myosin-IIA complex

**DOI:** 10.1186/1472-6807-13-31

**Published:** 2013-11-20

**Authors:** Udupi A Ramagopal, Natalya G Dulyaninova, Kristen M Varney, Paul T Wilder, Sridevi Nallamsetty, Michael Brenowitz, David J Weber, Steven C Almo, Anne R Bresnick

**Affiliations:** 1 Department of Biochemistry, Albert Einstein College of Medicine, 1300 Morris Park Avenue, Bronx, NY 10461, USA; 2 Department of Biochemistry and Molecular Biology, University of Maryland School of Medicine, 108 North Greene Street, Baltimore, MD 21201, USA; 3 Current address: Division of Biological Sciences, #4 16th Cross Sadshivanagar, Bangalore 560080, India

**Keywords:** X-ray crystallography, NMR, S100A4, Myosin-II, Cytoskeleton, Coiled-coil

## Abstract

**Background:**

S100A4, a member of the S100 family of Ca^2+^-binding proteins, modulates the motility of both non-transformed and cancer cells by regulating the localization and stability of cellular protrusions. Biochemical studies have demonstrated that S100A4 binds to the C-terminal end of the myosin-IIA heavy chain coiled-coil and disassembles myosin-IIA filaments; however, the mechanism by which S100A4 mediates myosin-IIA depolymerization is not well understood.

**Results:**

We determined the X-ray crystal structure of the S100A4Δ8C/MIIA^1908-1923^ peptide complex, which showed an asymmetric binding mode for the myosin-IIA peptide across the S100A4 dimer interface. This asymmetric binding mode was confirmed in NMR studies using a spin-labeled myosin-IIA peptide. In addition, our NMR data indicate that S100A4Δ8C binds the MIIA^1908-1923^ peptide in an orientation very similar to that observed for wild-type S100A4. Studies of complex formation using a longer, dimeric myosin-IIA construct demonstrated that S100A4 binding dissociates the two myosin-IIA polypeptide chains to form a complex composed of one S100A4 dimer and a single myosin-IIA polypeptide chain. This interaction is mediated, in part, by the instability of the region of the myosin-IIA coiled-coil encompassing the S100A4 binding site.

**Conclusion:**

The structure of the S100A4/MIIA^1908-1923^ peptide complex has revealed the overall architecture of this assembly and the detailed atomic interactions that mediate S100A4 binding to the myosin-IIA heavy chain. These structural studies support the idea that residues 1908–1923 of the myosin-IIA heavy chain represent a core sequence for the S100A4/myosin-IIA complex. In addition, biophysical studies suggest that structural fluctuations within the myosin-IIA coiled-coil may facilitate S100A4 docking onto a single myosin-IIA polypeptide chain.

## Background

S100A4 is a member of the S100 family of small, dimeric, EF-hand proteins. The S100 proteins, of which there are 21 family members in humans, primarily function as Ca^2+^-sensors to modulate a multitude of biological processes. The majority of S100 proteins are symmetric dimers characterized by the presence of two EF-hand Ca^2+^-binding loops per subunit; an N-terminal pseudo EF-hand (EF1) comprising 14 residues and a C-terminal canonical EF-hand (EF2) comprising 12 residues [1,2]. Calcium-binding to the C-terminal EF-hand induces a structural rearrangement involving the reorientation of helix 3 relative to helix 4, resulting in the exposure of a hydrophobic cleft that forms the binding surface for target proteins [3]. As a consequence, target binding for most S100 proteins is strictly Ca^2+^-dependent.

The reported equilibrium dissociation constants (K_D_) for the S100A4/Ca^2+^ interaction are relatively weak in the absence of target (^Ca^K_D_ = 54 μM, EF1; ^Ca^K_D_ = 3.3 μM, EF2) and increase approximately 10-fold in the presence of target (^Ca^K_D_ = 3.6 μM, EF1; ^Ca^K_D_ = 0.26 μM, EF2) [4]. Studies with other S100 proteins suggest that the increased Ca^2+^-binding affinity observed in the presence of target is due to a ligand-induced reduction in S100 backbone and side chain dynamics [5,6]. Localized target-mediated enhancement of the S100A4/Ca^2+^ interaction would permit high intracellular S100A4 expression levels (e.g. 3 – 5 μM [7]) without the depletion of free intracellular Ca^2+^ levels and the disruption of Ca^2+^ oscillations. Proteomic and localization studies have shown that S100A4 is enriched in the pseudopodia of migrating cells [8-10]. Moreover a S100A4 biosensor, which reports on Ca^2+^-bound S100A4, has shown that activated S100A4 localizes to the leading edge of polarized, migrating cells [11,12]. The enrichment of S100A4 in protrusive structures is consistent with cell-based functional studies demonstrating that S100A4 expression modulates the migratory capacities of a broad range of cell types [7,9,13,14]. Consistent with a role in regulating cell motility, S100A4 has a number of reported cytoskeletal and scaffold protein targets including non-muscle tropomyosin 2, liprin-β1 and non-muscle myosin-IIA [15-17].

Non-muscle cells can express three myosin-II heavy chain isoforms (NMHC-IIA – MYH9, NMHC-IIB – MYH10, NMHC-IIC – MYH14), which exhibit 64-80% amino acid identity [18]. Each heavy chain is comprised of an N-terminal motor domain that contains the ATP- and actin-binding sites, an intermediate rod domain that dimerizes to form an α-helical coiled-coil and a C-terminal tailpiece. Despite the fairly high sequence conservation between the non-muscle myosin-II isoforms, studies with assembly-competent myosin-II rod constructs, which lack the myosin-II motor domain, demonstrate that S100A4 preferentially recognizes and disassembles myosin-IIA rods [19,20]. Although recent reports indicate that S100A4 binds with high affinity to isolated myosin-IIC peptides, S100A4-mediated regulation of myosin-IIC assembly has not yet been examined [21]. For myosin-IIA, studies in mammary adenocarcinoma cells have shown that the S100A4/myosin-IIA interaction regulates the placement of pseudopodial protrusions during chemotactic migration [9]. In addition, macrophages with a genetic deletion for S100A4 exhibit a myosin-IIA overassembly defect that results in impaired cellular protrusions and defective chemotaxis [7]. Together these data support a role for the S100A4/myosin-IIA interaction in modulating pseudopodial structures, and as a consequence, chemotaxis. Consistent with its function in modulating cellular motility, S100A4 overexpression enhances the invasive capabilities and metastatic dissemination of tumor cells [22-25].

We report the structure of the Ca^2+^-S100A4/myosin-IIA complex, which demonstrates an unusual mode of S100 protein target recognition, and is in accordance with the recently described X-ray and NMR structures of S100A4 bound to longer myosin-IIA peptides [21,26]. In addition, biophysical studies of the Ca^2+^-S100A4/myosin-IIA complex suggest that instability of the myosin-IIA coiled-coil is a contributing factor to S100A4-mediated myosin-IIA depolymerization.

## Results

### Interaction of S100A4 with myosin-IIA

**Figure 1:**
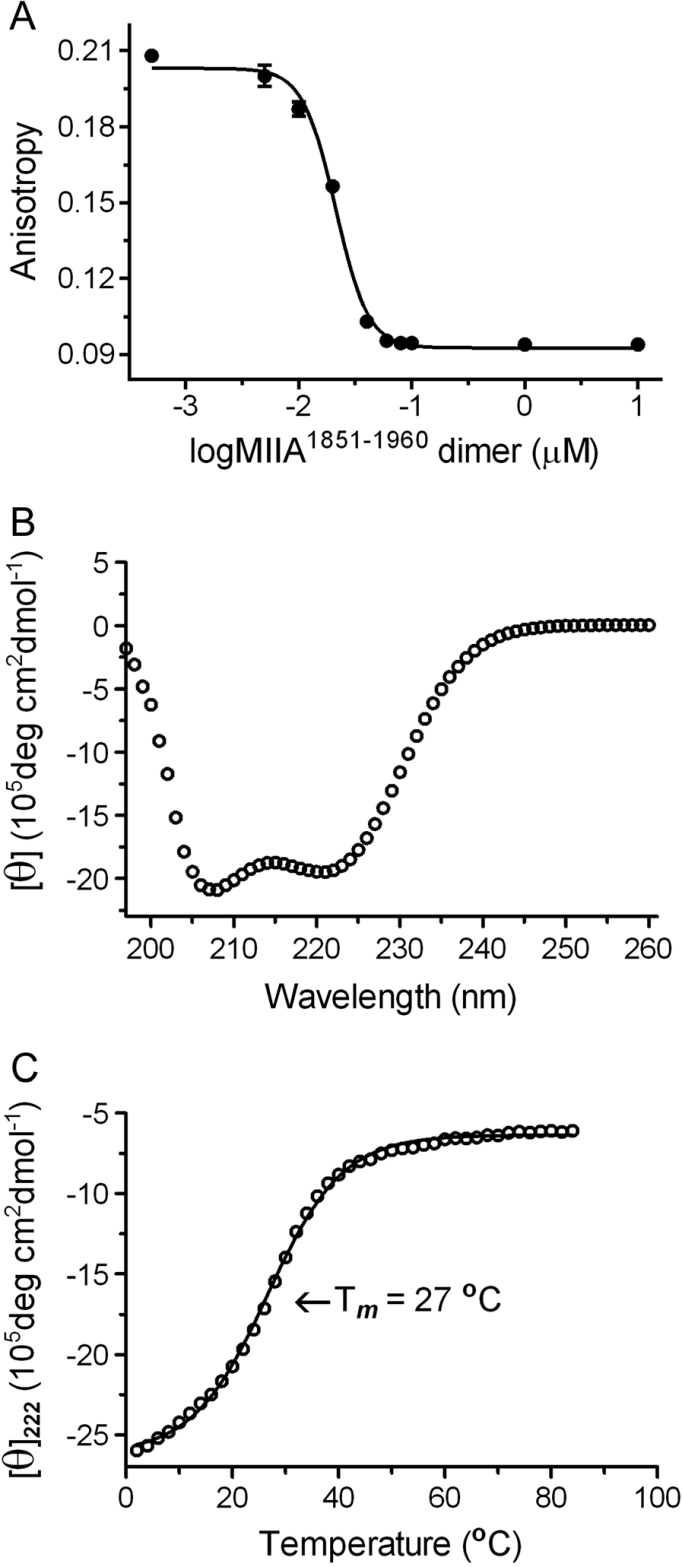
**Properties of MIIA**^**1851-1960**^**. (A)** Competition fluorescence anisotropy measurements of S100A4 binding to MIIA^1851-1960^, IC_50_ = 21.2 ± 2.0 nM. Values represent the mean ± standard deviation from two independent experiments. **(B)** Far-UV spectra of 40 μM MIIA^1851-1960^ monomer. **(C)** The thermal stability of MIIA^1851-1960^ (40 μM monomer concentration) was assayed by monitoring the ellipticity at 222 nm. Data represent the average of two independent experiments.

Our previous studies showed that S100A4 binds the MIIA^1904-1927^ peptide with a stoichiometry of one peptide per S100A4 dimer [27]. To examine the interaction of S100A4 with a longer, more physiologically-relevant myosin-IIA construct, we used MIIA^1851-1960^, which contains approximately 75 residues from the myosin-IIA coiled-coil and the entire C-terminal tailpiece, and encompasses the myosin-II extended assembly competence domain [28,29]. MIIA^1851-1960^ inhibits binding of the MIIA^1904-1927^ peptide to S100A4 with an IC50 of 21.2 ± 2.0 nM (Figure 1A). Circular dichroism spectroscopy of MIIA^1851-1960^ demonstrated that the peptide is primarily α-helical as evidenced by characteristic minima at 222 and 208 nm (Figure 1B). An examination of the thermal stability of MIIA^1851-1960^ revealed a relatively modest transition midpoint (T_m_) of 27°C for unfolding (Figure 1C), which was reversible (data not shown). The dimeric state of MIIA^1851-1960^ was confirmed by sedimentation equilibrium studies performed at 22°C (Table 1, Additional file 1: Figure S1). While our thermal denaturation studies suggest some MIIA^1851-1960^ monomer may be present, we did not detect any monomer in sedimentation equilibrium studies which were performed at higher protein concentrations than the thermal stability assays.

**Table 1:** Summary of sedimentation equilibrium data

**MIIA**^**1851-1960**^**: S100A4**^**a**^	**Predicted MW (Da)**^**b**^	**Experimental MW (Da)**
MIIA^1851-1960^	24,795	25,651 ± 308
0.5 MIIA:1.0 S100A4	35,592	35,271 ± 425
1.0 MIIA:1.0 S100A4	32,893	32,918 ± 490

^a^dimer: dimer ratio.

^b^Predicted molecular weights are based on sequence, which were confirmed by mass spectrometry; S100A4 dimer – 23,194.6 g/mol; MIIA^1851-1960^ monomer – 12,397.5 g/mol.

Sedimentation equilibrium studies of the S100A4/MIIA^1851-1960^ complex using a 0.5:1 molar ratio of MIIA^1851-1960^ dimer to S100A4 dimer revealed a species with a weight-average molecular mass of 35,271 ± 425 Da, which is consistent with the predicted mass of a S100A4 dimer with a single bound MIIA^1851-1960^ polypeptide chain (35,592 Da) (Table 1). At a 1:1 molar ratio of MIIA^1851-1960^ dimer to 1 S100A4 dimer we observed a weight-average molecular mass of 32,918 ± 490 Da, which likely corresponds to a mixture of S100A4 dimers with a single bound MIIA^1851-1960^ polypeptide chain and MIIA^1851-1960^ dimers (Table 1).

**Figure 2:**
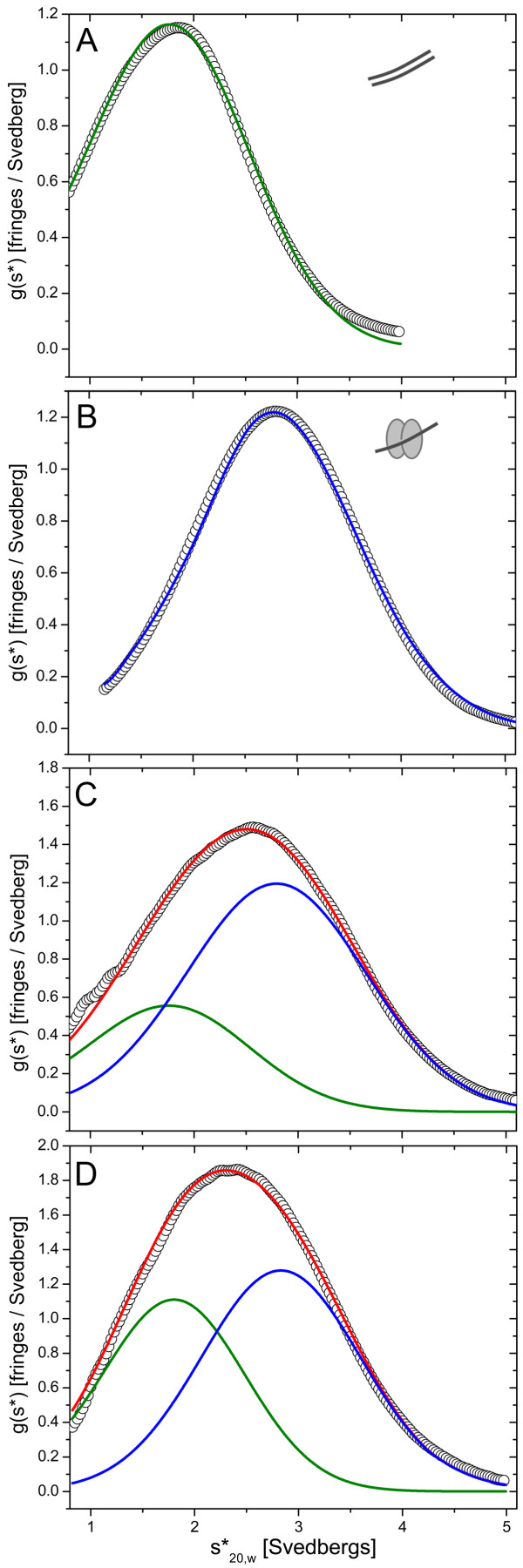
**Sedimentation velocity of the S100A4/MIIA**^**1851-1960 **^**complex.** Plots of sedimentation coefficient distribution g*(s) versus S_20,w_ for MIIA^1851-1960^ alone **(A)** and MIIA^1851-1960^/S100A4 mixtures at molar ratios of 0.5:1 **(B)**, 1:1 **(C)**, and 1.5:1 **(D)** of MIIA^1851-1960^ dimer:S100A4 dimer, which are represented by the open circles. The green line is the best fit to MIIA^1851-1960^, the blue line is the best fit to the S100A4 dimer/MIIA^1851-1960^ monomer complex and the red line is the best fit for all species in the S100A4/MIIA^1851-1960^ mixtures.

Given the similarity in the molecular weights of the S100A4 and MIIA^1851-1960^ dimers, the S100A4/MIIA^1851-1960^ complex was further evaluated in sedimentation velocity experiments where MIIA^1851-1960^ was added to S100A4 at increasing molar ratios. The time derivative plots for MIIA^1851-1960^ alone (Figure 2A) and S100A4 alone (Additional file 1: Figure S2) showed that each protein sedimented as a single homogenous species with S values of 1.91 S and 1.9 S, respectively (Additional file 1: Table S1). At a molar ratio of 0.5:1 MIIA^1851-1960^ dimer to S100A4 dimer there was a shift in the S value to 2.87 S consistent with the formation of an S100A4/MIIA^1851-1960^ complex (Figure 2B). At increasing molar ratios of MIIA^1851-1960^ dimer to S100A4 dimer (1:1, 1.5:1), the best fit for the time derivative plot was to a two-component model. This analysis indicated a peak at 2.89 S that corresponded to the S100A4/MIIA^1851-1960^ complex and a second peak at 1.9 S, which was consistent with the presence of unperturbed MIIA^1851-1960^ dimer (Figure 2C and 2D, Additional file 1: Table S1). The 2.89 S species had an estimated mass of 31.5 ± 0.2 kDa. Given the thermal instability of MIIA^1851-1960^ and our observation that S100A4 forms a thermostable dimer at submicromolar concentrations [4,27,30], the 2.89 S species likely corresponds to a S100A4 dimer bound to a single MIIA^1851-1960^ polypeptide (calculated mass of 35,592 Da), which is consistent with our previous chemical cross-linking studies [27].

### Characterization of S100A4Δ8C

**Figure 3:**
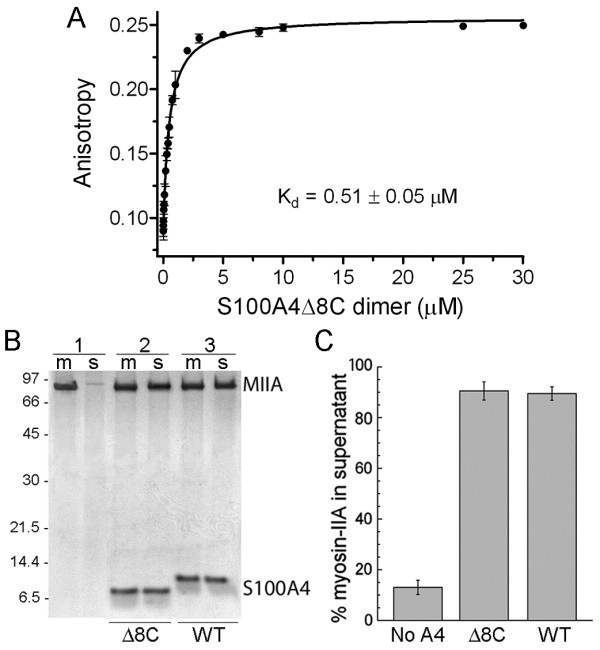
**S100A4Δ8 exhibits comparable myosin-IIA binding activity to wild-type S100A4. (A)** Fluorescence anisotropy measurements of S100A4Δ8 binding to FITC-MIIA^1904-1927^. Values represent the mean ± standard deviation from two independent experiments. A K_D_ of 0.51 ± 0.05 μM was determined from the fit to a single site saturation binding curve. **(B)** Representative gel of myosin-IIA disassembly assays performed at a ratio of 1:1 S100A4 dimer:myosin-IIA rod. 1 – myosin-IIA in the absence of S100A4; 2 – myosin-IIA in the presence of S100A4Δ8; 3 – myosin-IIA in the presence of wild-type S100A4 (m = reaction mixture, s = supernatant). **(C)** Quantification of disassembly assays. Values represent the mean ± standard deviation from two independent experiments.

Since we were unable to crystallize the wild-type full-length S100A4 with myosin-IIA peptides and our previous structural studies demonstrated that at high protein concentrations residues Glu91-Gln97 mediate interactions between symmetry-related S100A4 dimers in the crystalline state [4], we created a series of S100A4 C-terminal truncations (S100A4Δ3C, S100A4Δ4C, S100A4Δ6C, S100A4Δ7C, S100A4Δ8C, S100A4Δ9C and S100A4Δ13C; where “Δ-number” represents the number of residues deleted from the S100A4 C-terminus) that were expected to reduce S100A4 self-association. The S100A4Δ8C construct was used since it exhibited minimal self-association in the myosin-IIA peptide-bound state as assessed by dynamic light scattering and NMR. The ability of S100A4Δ8C to bind myosin-IIA was assessed in an anisotropy assay using FITC-labeled MIIA^1904-1927^, which binds wild-type S100A4 with a K_D_ of 0.26 ± 0.03 μM [27]. The measured dissociation constant for S100A4Δ8C (0.51 ± 0.05 μM) was comparable to that observed for the wild-type S100A4 (Figure 3A). In addition, binding was Ca^2+^-dependent as no binding was observed in the presence of EGTA (data not shown). Using assembly competent myosin-IIA rods (MIIA^1339−1960^), we monitored the ability of S100A4Δ8C to disassemble preformed myosin-IIA filaments. At a molar stoichiometry of one S100A4Δ8C dimer per myosin-IIA rod, S100A4Δ8C disassembled approximately 85% of the myosin-IIA filaments, which was similar to the disassembly observed in the presence of wild-type S100A4 (Figure 3B and 3C).

### X-ray Structures of S100A4Δ8C and the S100A4Δ8C/MIIA^1908-1923^ peptide complex

**Figure 4:**
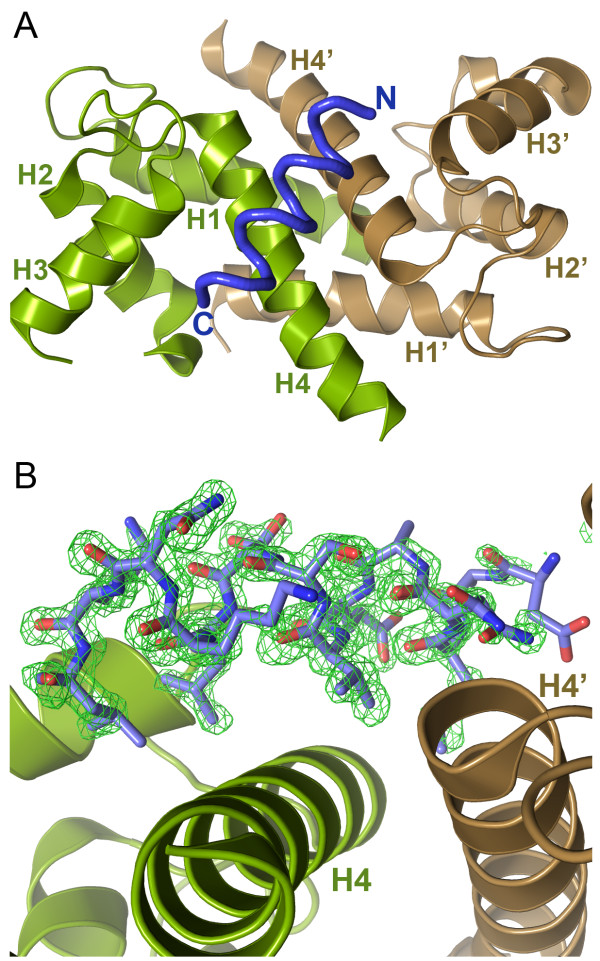
**Structure of the S100A4Δ8C/MIIA**^**1908-1923**^** complex. (A)** Ribbon diagram of the S100A4Δ8C/MIIA^1908-1923^ complex showing the S100A4 subunits in green (subunit A) and brown (subunit B) and the MIIA^1908-1923^ peptide in blue. **(B)** Refined model of the S100A4Δ8C/MIIA^1908-1923^ peptide structure. The myosin-IIA peptide (residues Asp1908-Leu1917) is shown in stick representation superimposed onto the F_o_-F_c_ electron density map, contoured at 3.0*σ*.

Both the S100A4Δ8C and S100A4Δ8C/MIIA^1908-1923^ peptide structures were determined by molecular replacement using the native Ca^2+^-S100A4 structure (2Q91) truncated at Glu91 as the search model [4] (Table 2). For the apo-S100A4Δ8C structure there were four molecules in the asymmetric unit (two S100A4Δ8C dimers), with continuous density from residues Cys3-Gly92 for all molecules. Residues in the favored, allowed and disallowed regions in the Ramachandran plot account for 97.4%, 2.0% and 0.6% of the total residues, respectively. The S100A4Δ8C/MIIA^1908-1923^ structure contained a single S100A4 dimer in the asymmetric unit with the MIIA^1908-1923^ peptide bound asymmetrically across the dimer interface. The electron density was continuous from residues Ala2-Gly92 for both molecules in the asymmetric unit, except for residues Gly47-Arg49 (loop region between helix 2 and 3) of subunit A, which were not modeled. Continuous difference density was observed in difference Fourier syntheses (using F_o_-F_c_ coefficients; contoured at 3*σ*) following the first round of refinement, indicating the presence of a highly ordered peptide, asymmetrically bound to the S100A4 dimer (Figure 4A and 4B). Residues in the favored, allowed and disallowed regions in the Ramachandran plot account for 96.8%, 2.2% and 1.2% of the total residues, respectively. The two residues in the disallowed region, which deviate slightly from expected values, are Arg49 (subunit B) from the disordered region and the C-terminal Gly92.

**Table 2:** Crystallographic data and refinement statistics

	**S100A4Δ8C**	**S100A4Δ8C/MIIA**^**1908-1923**^
PDB ID	4HSZ	4ETO
Source	X29, BNL	X4A, BNL
Wavelength (Å)	1.075	0.979
Resolution limits (Å)	46.9 – 2.25	50 – 1.54
Space group	P1	P2_1_
Unit cell (Å) *a, b, c and* α, β, γ *(°)*	a = 28.80, b = 34.36, c = 95.31 and α = 95.48, β = 95.30, γ = 114.82	a = 30.28, b = 91.99, c = 32.86 and β = 112.6
Number of observations	31076	87461
Number of unique reflections	14935	24461
^a^Completeness (%)	96.4 (94.1)	99.6 (100)
Mean I/σI	19.6 (2.4)	27.8 (2.76)
^b^R_merge_ on I	6.3 (42.2)	6.3 (49.6)
B_wilson_ (Å^2^)	30.023	18.4
Refinement statistics		
Resolution limits (Å)	46.9 – 2.25	46.0 – 1.54
Number of reflections (work/free)	14159/750	23168/1247
Cutoff criteria I/σI	0	0
Protein/water atoms	2904/9	1530/86
R_work_/R_free_ (5% of data)	0.228/0.277	0.209/0.251
^c^Bonds (Å)/angles (°)	0.007/0.984	0.020/1.823
Mean B (Å^2^)	56.2	20.14

^a^Parentheses indicate statistics for the high–resolution data bin for x–ray and refinement data.

^b^*R*_merge_ = ∑*hkl* ∑*i*|*I*(*hkl*)*i* – < *I*(*hkl*) > |/∑*hkl*∑*i* < *I*(*hkl*)*i* >.

^c^Values indicate root–mean–square deviations in bond lengths, bond angles, and B-factors of bonded atoms.

**Figure 5:**
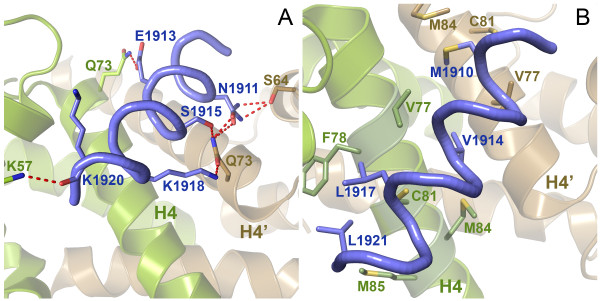
**Interactions within the S100A4Δ8C/MIIA**^**1908-1923**^** complex.** Binding of the MIIA^1908-1923^ peptide (blue) to the S100A4Δ8C dimer involves direct electrostatic interactions **(A)** and water mediated electrostatic interactions (not shown) as well as hydrophobic interactions **(B)**. Hydrogen bonds are shown as red dotted lines.

The 1.54 Å structure of the S100A4/MIIA^1908-1923^ complex revealed a single MIIA^1908-1923^ peptide binding across the S100A4 dimer interface (Figure 4A). Electron density was observed only for residues Asp1908-Leu1921 of the MIIA^1908-1923^ peptide. As shown in Figure 5A, there is an extensive H-bond network between myosin-IIA residues Asn1911, Ser1915 and Lys1918, and Ser64 and Gln73 on subunit B. In addition peptide residues Glu1913 and Lys1920 form H-bonds with Gln73 and Lys57 of subunit A. Met1910, Val1914, Leu1917 and Leu1921, which correspond to the *a* and *d* positions of the myosin-IIA coiled-coil (*a* – Val1914, Leu1921; *d* – Met1910, Leu1917), also partcipate in S100A4 binding. Met1910 and Val1914 intercalate between helices 4 and 4′ at the S100A4 dimer interface, while Leu1917 and Leu1921 insert into the hydrophobic cleft of subunit A (Figure 5B).

The total buried surface area upon complex formation is appproximately 1348 Å^2^, where 684 Å^2^ and 664 Å^2^ are contributed by the MIIA^1908-1923^ peptide and S100A4, respectively. Overall, nearly 380 contacts with a separation of less than 5.0 Å and 8 hydrogen bonds are observed at the myosin-IIA peptide and S100A4 interface. The calculated rms deviations for Cα atoms for residues 3–86 from the Ca^2+^-bound S100A4 (2Q91) with the Ca^2+^-bound S100A4Δ8C (4HSZ), the S100A4Δ8C/MIIA^1908-1923^ peptide complex (4ETO) and the previously reported S100A4/MIIA^1893-1935^ complex (3ZWH) were 1.2 Å, 1.3 Å and 1.1 Å, respectively; indicating that neither myosin-IIA binding, nor the C-terminal truncation, alters the overall conformation of the S100A4 dimer. The most significant differences were observed in the loop connecting helices 2 and 3 (residues 45–52).

To examine myosin-IIA peptide binding to S100A4Δ8C in solution, the MIIA^1908-1923^ peptide used in crystallization studies was titrated into a ^15^N-labeled sample of Ca^2+^-bound S100A4Δ8C. A comparison of these data to a similar titration performed with wild-type S100A4 [4] revealed that of the 44 correlations shown previously to shift upon MIIA^1908-1923^ binding to wild-type S100A4, 34 of these chemical shift perturbations were observed in titrations with S100A4Δ8C. These included residues in helix 1 (Cys3 Met12, Val13, Phe16, and Lys18), the pseudo-EF-hand (Gly21 Asn30, Lys31), the hinge (Arg40, Glu41, Leu42, Arg49, Thr50, and Asp51), helix 3 (Glu52, Phe55, Asn56, Leu58, Met59, Ser60 and Leu62), the typical EF-hand (Asp63, Ser64, Asn68, Gln73, and Glu74), helix 4 (Cys76, Val77, Met85, and Cys86), and the C-terminal tail (Asn87, Gln97). Following the addition of the MIIA^1908-1923^ peptide, differences in chemical shift were also observed in the ^1^H-^15^N correlations for Ser14, His17, Ser20, Phe27, Gly47, Leu79, Ala83, Glu88, Phe89, Gly92 and for Phe93, but these changes did not occur or were not the same as perturbations found in titrations with wild-type S100A4. In addition, there were fewer overlapping ^1^H-^15^N correlations for the S100A4Δ8C/peptide complex since correlations arising from residues 94–101 were absent in this S100A4 construct. Overall, these NMR data confirm that S100A4Δ8C binds the MIIA^1908-1923^ peptide in a very similar structure/orientation in solution as observed for wild-type S100A4.

### Crystal packing interactions

**Figure 6:**
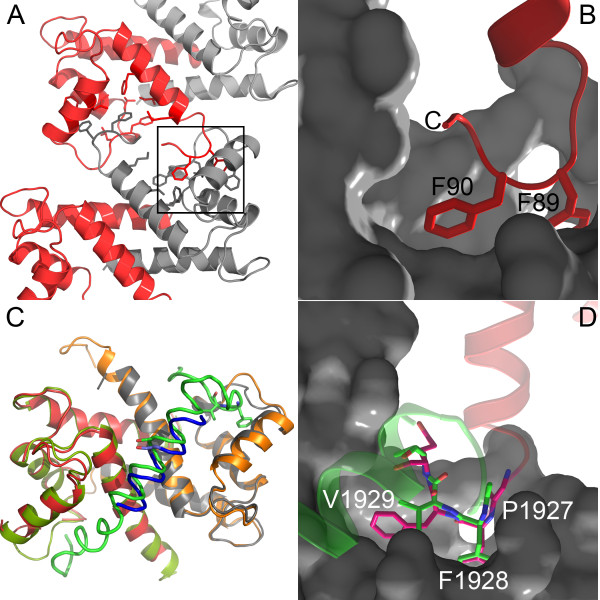
**Self-association of S100A4Δ8 molecules in the crystal lattice. (A)** The Ca^2+^-S100A4Δ8 dimer is shown with one red and one gray subunit. The S100A4Δ8C dimers in the crystal are positioned such that the C-terminal tail of subunit B (red) inserts into the hydrophobic cleft (subunit A-gray) of the adjacent S100A4 dimer. Similarly, the C-terminal tail of subunit A (gray) from the same adjacent dimer inserts into the hydrophobic cleft on subunit B (red). **(B)** High magnification view of the boxed area in **(A)**. Phe89 and Phe90 of the C-terminal tail are buried inside the hydrophobic cleft of the symmetry-related molecule. **(C)** Superimposition of the S100A4/MIIA^1893-1935^ structure (3ZWH; peptide – green, S100A4 dimer – orange and pistachio) onto the S100A4Δ8C/MIIA^1908-1923^ structure (peptide – blue, S100A4Δ8C dimer – gray and red). **(D)** Myosin-IIA residues Pro1927-Val1930 (green) from the S100A4/MIIA^1893-1935^ structure (3ZWH) occupy the same region of the hydrophobic cleft as the C-terminal tail of S100A4Δ8C. S100A4Δ8C residues Glu88-Glu91 (magenta) and myosin-IIA residues Pro1927-Val1930 (green) adopt a similar conformation. Note that the S100A4 (red) and myosin-IIA (green) helices approach the hydrophobic cleft from opposite orientations.

An examination of the crystal packing interactions for S100A4Δ8C shows that the C-terminal tail (residues Glu88-Glu91) of subunit A interacts with the hydrophobic cleft formed between helices 3 and 4 of the crystallographically related subunit B (Figure 6A) and vice versa. These interactions result in the formation of rods of S100A4 dimers running parallel to the crystallographic *b-axis* for the AB subunits and parallel to the *ab* diagonal for the CD subunits (Additional file 1: Figure S3). The inter-dimer contacts between the C-terminal tail of one S100A4 dimer and the target binding cleft of the symmetry related molecule are primarily mediated by Phe89 and Phe90 of the C-terminal tail, and residues Phe45, Leu46, Phe55, and Leu58 in the hinge and helix 3 of the symmetry related molecule (Figure 6B). A comparison of these S100A4 inter-dimer interactions with the S100A4/MIIA^1893-1935^ structure (3ZWH, Figure 6C) revealed that residues Pro1927-Val1930 of the myosin-IIA peptide adopt a similar type I β-turn as S100A4 residues Glu88-Glu91, with Phe1928 and Val1929 residing deep within the hydrophobic cavity formed by the hinge and helix 3 (Figure 6D). Although the orientation of the S100A4 C-terminal helix (Figure 4D, red) as it approaches the hydrophobic cleft is almost opposite to that of the myosin-IIA peptide (Figure 4D, green), the Cα atoms for Glu88-Glu91 of S100A4 and Pro1927-Val1930 of myosin-IIA have an overall rms deviation of 0.921 Å; demonstrating that when bound in the hydrophobic cleft, the C-terminal tail of S100A4 and myosin-IIA present similar geometric and chemical features.

### Solution studies of a larger S100A4/MIIA^1893-1923^ peptide complex

**Figure 7:**
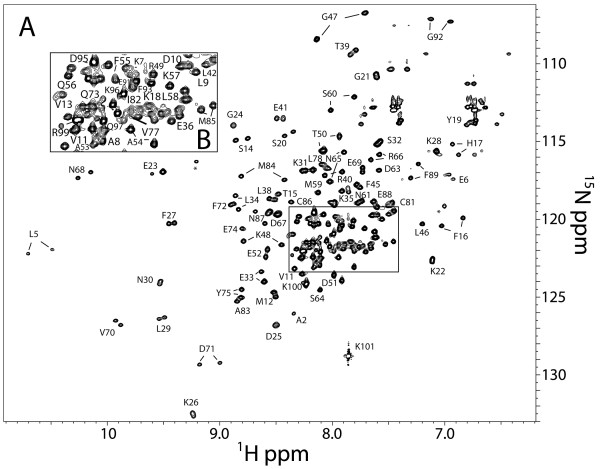
**NMR assignments for MIIA**^**1893-1923**^**-bound Ca**^**2+**^**-S100A4. (A)** A ^1^H-^15^N heteronuclear single quantum coherence spectrum (HSQC) of MIIA^1893-1923^-bound S100A4 shown together with the resonance assignments. **(B)** Insert of the HSQC spectrum illustrating the assignments for the most crowded region of the HSQC spectrum. The two weak contours labeled with an asterisk (*) were not assigned since they did not have observable correlations to any other residues.

Since MIIA^1893-1923^ bound S100A4 with a higher affinity than MIIA^1908-1923^[4], and exhibited better spectral properties than MIIA^1908-1923^, we examined this longer peptide in NMR studies to further investigate the solution properties of the asymmetric binding mode observed for myosin-IIA in the S100A4Δ8C/MIIA^1908-1923^ complex. As expected for a complex with a dissociation constant in the nanomolar range, MIIA^1893-1923^-bound S100A4 exhibited resonances with slow exchange on the chemical shift timescale. This property resulted in spectra exhibiting narrow linewidths, which is consistent with little, if any, broadening contributions from intermediate exchange. The ^1^H-^15^N HSQC of the Ca^2+^-S100A4/MIIA^1893-1923^ complex showed that peak doubling occurred for approximately two-thirds of the observable ^1^H-^15^N correlations throughout all regions of the protein (Figure 7). The resonance doubling complicated the assignment of backbone and side chain resonances, but it was still possible to sequence-specifically assign all of the observed correlations using standard NMR techniques. The observed peak doubling is consistent with the binding mode of the shorter MIIA^1908-1923^ peptide in our X-ray structure, in which peptide binding breaks the symmetry of the S100A4 dimer interface that is observed in the Ca^2+^-bound S100A4, resulting in two distinct electronic shielding environments for several residues.

**Figure 8:**
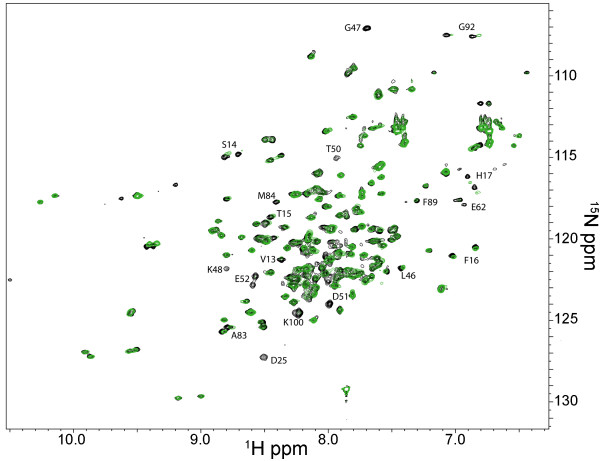
^**1**^**H-**^**15**^**N heteronuclear single quantum coherence spectrum (HSQC) of full-length Ca**^**2+**^**-S100A4 at 600 MHz.** Black: S100A4 bound to the MIIA^1893-1923^ peptide (0.25 mM S100A4 subunit and 0.75 mM MIIA^1893-1923^). Green: Following the addition of 42 μM MIIA^1893-1923^-Cys-Proxyl peptide. Residues exhibiting a significant reduction in intensity are labeled.

**Figure 9:**
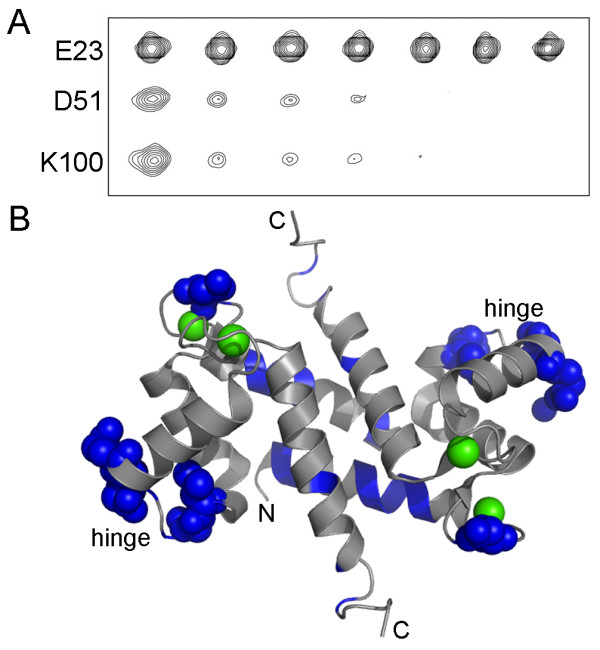
**Paramagnetic relaxation effects observed for **^**1**^**H-**^**15**^**N HSQC correlations of Ca**^**2+**^**-S100A4 in a titration with a MIIA**^**1893-1923 **^**peptide spin-labeled at its C-terminus. (A) **^1^H-^15^N HSQC correlations for Glu23, Asp51, and Lys100 of Ca^2+^-bound S100A4 in the presence of increasing amounts of spin-labeled MIIA^1893-1923^ peptide (0, 2.6, 5.3, 11, 21, 42, and 95 μM). **(B)** Ribbon diagram of Ca^2+^-bound S100A4 (PDB 2Q91) with residues affected by the spin label highlighted in blue. Residues exhibiting the strongest effects are shown as spheres. Arg99 and Lys100 are not shown on the ribbon diagram since these residues are not observed in the X-ray structure due to the mobility of the C-terminal loop [4].

A comparison of the ^1^H-^15^N HSQC spectra of the S100A4/MIIA^1893-1923^ complex (peak doubling) to that of the Ca^2+^-S100A4 (no peak doubling) revealed that it was not possible to analyze the chemical shift perturbations due to the ambiguities that peak doubling introduced. To address this problem, a Proxyl spin-label was covalently attached to the C-terminus of the MIIA^1893-1923^ peptide and the distance-dependent effects (1/r^6^) of paramagnetic relaxation were mapped onto the Ca^2+^-S100A4 structure (Figure 8) [31]. At the lowest concentration of MIIA^1893-1923^-Cys-Proxyl peptide (2.6 μM), large reductions in cross-peak intensities were observed for residues in loop 1 (Asp25), the beginning of helix 3 (Asp51, Glu52) and in the C-terminal loop (Lys100). These relaxation effects, plus those observed at the next titration point (5.3 μM), were consistent with the C-terminus of the MIIA^1893-1923^ peptide being located near residues in helix 3 and the hinge (Leu46, Gly47, Asp51, Glu52; Figure 9A and 9B). Although the cross-peak intensities for residues Arg99 and Lys100 were also significantly reduced by low MIIA^1893-1923^-Cys-Proxyl peptide concentrations, their exact positions in the Ca^2+^-S100A4 structure were uncertain since the C-terminal loop is mobile in solution. In addition, these residues were not observed in the Ca^2+^-S100A4 X-ray structure [4], which is consistent with high structural plasticity and dynamic behavior. Other residues in Ca^2+^-S100A4 were affected to a lesser degree, and only at higher concentrations of the spin-labeled peptide (i.e. ≥ 21 μM), including residues in helix 1 (Leu5, Glu6, Val13, Ser14, and His17), loop 1 (Lys26), the hinge (Lys48), and helix 3 (Thr50). However, data at higher spin-labeled peptide concentrations were interpreted cautiously because of the potential for outer sphere effects from free spin-labeled peptide [31-33]. Altogether, these solution data support the asymmetric binding mode for the myosin-IIA peptide and indicate that the peptide can bind in either of two orientations across the S100A4 dimer interface.

## Discussion

**Figure 10:**
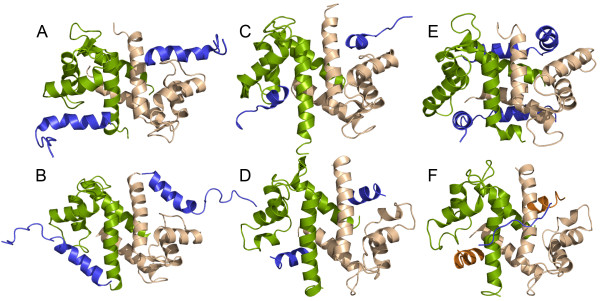
**Comparison of six S100-target complexes. (A)** S100B-p53 (1DT7); **(B)** S100B-NDR kinase (1PSB); **(C)** S100A1-TRTK12 (2KBM); **(D)** S100A10-annexin2 (1BT6); **(E)** S100A6-SIP (2JTT) and **(F)** S100A10-annexin2-AHNAK (4DRW). The S100 subunits are shown in green and tan, and the peptide ligand in blue. For the S100A10-annexin2-AHNAK ternary complex, the annexin2 peptide is shown in orange.

To date, structural studies of S100-target complexes have revealed a variety of binding modes with respect to target recognition, which are typically characterized by the formation of symmetric complexes with a 1:1 S100 subunit:target stoichiometry (Figure 10) [34-39]. For S100B and S100A1-target complexes, target binding is primarily mediated by interactions with the hydrophobic cleft formed by helices 3 and 4 of the S100 protein, although the orientation of the target peptide within the hydrophobic cleft can vary significantly [34-36]. Other target binding modes include interactions with helix 4 of one S100 subunit and helix 1′ of the second S100 subunit as observed for S100A10 and S100A11-target complexes [38,39], and in the case of the S100A6/SIP complex, target binding involves bidentate interactions with the hydrophobic cleft of one S100 subunit and helix 1′ of the second S100 subunit [37]. The structure of the S100A4Δ8C/MIIA^1908-1923^ peptide complex demonstrates a fourth binding mode, in which the myosin-IIA peptide binds asymmetrically across helices 4 and 4′ at the S100A4 dimer interface. Asymmetric target binding is emerging as a common feature amongst the S100 protein family as this binding mode is also observed for AHNAK bound to the S100A10/annexin A2 complex and SMARCA3 bound to the S100A10/annexin A2 complex [40-42].

The recent structures of S100A4 bound to longer myosin-IIA peptides have demonstrated that the N- and C-termini of the peptide insert into the hydrophobic clefts formed by helices 3 and 4 of each S100A4 subunit to mediate high affinity binding [21,26]; however, our structural studies indicate that a significantly shorter myosin-IIA peptide is sufficient to promote an asymmetric binding mode. A contributing factor to the asymmetric disposition of the myosin-IIA peptide is that it makes contacts with dimer-related residues on helices 4 and 4′ of each S100 subunit. In particular, Val77, Cys81 and Met84 of each S100A4 subunit form interactions with myosin-IIA that position the peptide nearly orthogonally across the dimer interface. Our previous biochemical studies support the contribution of these residues to myosin-IIA binding as substitution of Cys81 with Ala or Ser reduces S100A4 binding to myosin-IIA by 20- and 55-fold, respectively, and disrupts S100A4-mediated myosin-IIA disassembly [43].

In addition to crystallographic and biochemical data, NMR data supports a model in which residues 1908 to 1923 of the myosin-IIA heavy chain represent the core sequence for S100A4 recognition. In this regard, we examined whether this small peptide was sufficient to induce what is termed the “final folding” event that occurs upon S100 protein-target complex formation [6]. We examined whether conformational exchange in S100A4, which is observed in the absence of bound target, could be stabilized upon binding the MIIA^1908-1923^ peptide. As found for other wild-type and mutant S100 proteins [6,44], significant exchange broadening was observed in the Ca^2+^-bound S100A4Δ8C. Notably, this exchange broadening completely disappeared upon S100A4Δ8C/MIIA^1908-1923^ complex formation (data not shown). As with other S100 proteins, this behavior likely accounts for the higher Ca^2+^-binding affinity observed for S100A4 when bound to MIIA^1908-1923^[4]. This may be considered analogous to a “mini-folding” event in which target binding shifts the S100A4 equilibrium population from a highly dynamic, but weak Ca^2+^-binding state to a high affinity Ca^2+^ binding state that is composed of an S100A4 population with a narrower distribution of dynamic features. While the regulation of S100A4 protein dynamics upon target binding will be the subject of future studies, it is important to note that the MIIA^1908-1923^ peptide represents the core of the S100A4/myosin-IIA interaction since it is adequately sized and positioned to eliminate conformational exchange upon binding either S100A4Δ8C or wild-type protein [4]. Importantly, as most target-free S100 proteins have a relatively low affinity for Ca^2+^ (i.e., versus bound to target) this allows for numerous S100 proteins to exist at high cellular concentrations (> 1 μM) without significantly depleting intracellular [Ca^2+^]_free_ levels and short-circuiting Ca^2+^ oscillations. Thus, reasonably high S100A4 levels (e.g., 3–5 μM [7]) can be poised inside the cell as myosin-IIA becomes available for complex formation and downstream cytoskeletal modulation.

An interesting feature of the S100A4/myosin-IIA complex is that myosin-IIA residues contributing to the coiled-coil interface also mediate S100A4 binding. These observations suggest a mechanism in which S100A4 binding induces partial unwinding of the myosin-IIA coiled-coil, and thus promotes myosin-IIA filament disassembly. The relatively low Tm (45°C) of the myosin-IIA rod (residues 1102–1960) [45], and the relative instability of the coiled-coil region that encompasses the S100A4 binding site suggests that this region of the myosin-IIA coiled-coil likely undergoes significant thermally-driven structural fluctuations (Figure 1C) [21,45]. Modest fluctuations in the structure of the coiled-coil would allow initial docking of S100A4 onto a single myosin-IIA polypeptide chain followed by partial wrapping of the myosin-IIA heavy chain across the S100A4 dimer and further unzipping of the coiled-coil. The free myosin-IIA heavy chain would then be available for subsequent capture by a second S100A4 dimer. Although turbidity studies with short myosin-IIA rod constructs (residues 1712–1960 and 1761–1960) support a binding stoichiometry of one S100A4 dimer per myosin-IIA polypeptide chain [21,46]; sedimentation assays with longer myosin-IIA rod constructs (residues 1339–1960) have shown that a stoichiometry of one S100A4 dimer to two myosin-IIA polypeptide chains (one myosin-IIA heavy chain dimer) is sufficient to promote maximal filament disassembly [19,43]. While S100A4 binds with low nanomolar affinities to isolated myosin-IIA peptides and short, relatively unstable myosin-IIA rod constructs, binding to longer myosin-IIA rods or full-length myosin-IIA appears to be significantly weaker [19,47]. These observations suggest that the loss of energy due to myosin-IIA heavy chain unzipping can modulate S100A4 binding affinity, as a significant fraction of the binding energy must be used to drive the dissociation of the two polypeptide chains in the myosin-IIA rod. Future studies directed at examining the requirement for the binding of one versus two S100A4 dimers per myosin-IIA heavy chain dimer for coiled-coil unzipping, as well as the reduction in free energy that occurs during unzipping will require the use of longer and more stable myosin-IIA coiled-coil constructs.

This proposed model of S100A4-mediated myosin-II heavy chain unzipping is analogous to the binding of single-stranded DNA binding protein and ribosomal protein S1 to single-stranded DNA and RNA segments, respectively, that transiently form during thermal breathing of the nucleic acid base pairs [48,49]. While the detailed mechanisms of polymer unzipping associated with these nucleic acid binding proteins will be different due to their multi-domain nature and/or oligomeric state, these biological systems provide a conceptual framework for considering the mechanism of S100A4 coiled-coil unzipping.

## Conclusions

Direct visualization of the S100A4/myosin-IIA complex has defined the chemical determinants required for myosin-IIA binding and the overall organization of the complex. These studies have identified residues 1908–1923 of the myosin-IIA heavy chain as a core sequence for formation of the S100A4/myosin-IIA complex. Moreover, these studies highlight the potential role of structural dynamics within the myosin-IIA coiled-coil and S100A4 in mediating the Ca^2+^-dependent S100A4/myosin-IIA interaction.

## Methods

### S100A4 purification

For the production of the untagged, full-length protein, the codon-optimized human S100A4 sequence was subcloned into the Nde1/BamH1 sites of pET11b (Novagen). The S100A4Δ8C deletion mutant (deletion of the C-terminal eight amino acids) was subcloned into the Nde1/BamH1 sites of pET11b. BL21(DE3)* cells were transformed with the codon-optimized human wild-type or Δ8C S100A4 and cultures were grown in Luria broth at 37°C to an OD_600_ of 0.8-1.0. Protein expression was induced with 0.7 mM IPTG and the cultures were grown for an additional 18–22 hrs at 27°C. The cells were harvested at 8000 rpm for 10 min, and the cell pellets were resuspended in lysis buffer (50 mM Tris pH 7.5, 10% glycerol, 300 mM KCl, 5 mM DTT, 1 mM EDTA, 1 mM PMSF and 5 μg/ml each of chymostatin, leupeptin, and pepstatin). The cell lysates were frozen at −80°C, thawed on ice, and sonicated. Following centrifugation of the lysate at 30000 *g* for 30 min, ammonium sulfate was added to the supernatant to 43% saturation, the sample was stirred for 20 min on ice and centrifuged at 30000 *g* for 10 min. CaCl_2_ was added to the supernatant to a final concentration of 2 mM, and the sample was applied to a Phenyl-Sepharose column (GE Healthcare) equilibrated in buffer P (20 mM Tris pH 7.5, 2 mM CaCl_2_, 300 mM KCl, 5 mM DTT, 1 mM EDTA, and 0.02% NaN_3_) containing 25 g ammonium sulfate/100 ml. The column was washed with 3 column volumes of buffer P containing 25 g ammonium sulfate/100 ml and then with 3 column volumes of buffer P without ammonium sulfate, and S100A4 was eluted with 20 mM Tris pH 7.5, 5 mM EGTA, 2 mM DTT, 2 mM TCEP, 1 mM EDTA and 0.02% NaN_3_. Fractions containing S100A4 were pooled and dialyzed against 2.0 L of buffer Q (20 mM Tris pH 7.5, 2 mM DTT, 2 mM TCEP and 0.02% NaN_3_). The dialyzed pool was applied to a Hi Prep 16/10 QXL column (GE Healthcare) and the column washed with 3 column volumes of buffer Q followed by a 200 ml gradient of 0–0.5 M NaCl in buffer Q. The purified proteins were stored at −80°C. For crystallization, S100A4 was gel-filtered on a HiLoad Superdex 75 26/60 column (GE Healthcare) and concentrated using a Vivaspin 15R ultrafiltration spin column (Sartorius Stedium, Life Technology).

For NMR studies, ^15^N-labeled wild-type S100A4 was purified from bacterial cells grown in M9 media containing ^15^N NH_4_Cl as described previously [4].

### Myosin-IIA peptides

The FITC-Ahx-TETADAMNREVSSLKNKLRRGDLP-CONH_2_ (FITC-MIIA^1904-1927^), Ace-TETADAMNREVSSLKNKLRRGDLP-CONH_2_ (MIIA^1904-1927^) and Ace-DAMNREVSSLKNKLRR-CONH_2_ (MIIA^1908-1923^) peptides were synthesized by Bio-Synthesis Inc. to a purity of >95% as determined by HPLC and mass spectrometry. A 5 mM stock solution of FITC-MIIA^1904-1927^ was prepared in 20 mM Tris pH 7.5, 150 mM KCl, 1 mM DTT and 0.02% NaN_3_, aliquoted and stored at −80°C. The concentration of the FITC-MIIA^1904-1927^ peptide was determined using the extinction coefficient for FITC. The concentrations of the MIIA^1908-1923^ and MIIA^1904-1927^ peptides were determined by quantitative amino acid analysis (Keck Biotechnology Resource Laboratory at Yale University, New Haven, CT). A 25 mM stock solution of MIIA^1908-1923^ was prepared in 10 mM Tris pH 7.5, 10 mM NaCl, and 0.02% NaN_3_, aliquoted and stored at −80°C. For NMR studies, two peptides Ac-RRKLQRELEDATETADAMNREVSSLKNKLRR-CONH_2_ (MIIA^1893-1923^) and Ac-RRKLQRELEDATETADAMNREVSSLKNKLRRC-CONH_2_ (MIIA^1893-1923^-Cys), which contained an additional Cys on the C-terminus were made by solid-state peptide synthesis to a purity of >95% as determined by HPLC and mass spectrometry. Stock peptide solutions were prepared in metal free double distilled H_2_O and concentrations were determined by quantitative amino acid analysis (BioSynthesis Inc.).

### Myosin-IIA proteins

A codon-optimized C-terminal fragment of the human myosin-IIA heavy chain that corresponds to residues 1851–1960 (MIIA^1851-1960^) was cloned into the NdeI/EcoR1 sites of pET26b and expressed in BL21(DE3)* cells using TB autoinducing media. MIIA^1851-1960^ was purified by sequential chromatography on High Q Sepharose anion exchange (GE Healthcare) and Bio-Scale Ceramic Hydroxyapatite Type I (Bio-Rad) columns as described previously [4] and then gel-filtered on a Sephacryl S200 26/60 column (GE Healthcare). The MIIA^1851-1960^ protein concentration was determined using the Bradford protein assay (Bio-Rad) and a MIIA^1851-1960^ standard of known concentration. The concentration of the MIIA^1851-1960^ standard was determined by amino acid analysis (Keck Biotechnology Resource Laboratory at Yale University, New Haven, CT). The mass of MIIA^1851-1960^ was confirmed by mass spectrometry.

### Peptide spin labeling

For the MIIA^1893-1923^-Cys peptide, 25 μl of a 29.77 mM stock peptide solution was added to 92 μl of metal free (Chelex-100 treated) double distilled H_2_O, 20 μl 500 mM TES pH 7.2, 2 μl 500 mM EDTA and incubated with pre-washed immobilized TCEP disulfide reducing gel (Pierce) for >1 hour at room temperature. The beads containing the immobilized TCEP and other particulates were removed using a 0.45 μm Nanosep MF GHP centrifugal device (Pall Life Sciences) before the addition of 61 μl freshly prepared 50 mg/ml 3-maleimido-Proxyl (Sigma-Aldrich) in DMSO. Purification of the Proxyl-labeled peptide (MIIA^1893-1923^-Cys-Proxyl) was performed by separation of the unlabeled and labeled peptides on a C18 column using a 0-90% acetonitrile gradient and monitoring at 214 nm and 280 nm. The unlabeled peptide does not absorb at 280 nm, whereas the Proxyl-labeled peptide has an extinction coefficient of 500 M^-1^ cm^-1^ at 280 nm. The reaction was complete in 4 hours at room temperature and further incubation resulted in the production of second labeled form of the peptide, likely from the slower modification of methionine by the 3-maleimido- Proxyl [50]. The purified MIIA^1893-1923^-Cys-Proxyl peptide (>99%) was resuspended in D_6_-DMSO (Cambridge Isotopes Labs) and the peptide concentration was determined using the extinction coefficient of the Proxyl label.

### S100A4 crystallization

S100A4Δ8C was freshly dialyzed against 20 mM Tris pH 7.5, 150 mM KCl, 1 mM DTT, 0.02% NaN_3_, and 0.5 mM CaCl_2_ and clarified by ultracentrifugation at 80000 rpm for 10 minutes at 22°C in TLA 120.2 rotor. S100A4 protein concentrations were determined using the Bradford protein assay (Bio-Rad) and a S100A4 standard of known concentration. The concentration of the S100A4 standard was determined by quantitative amino acid analysis (Keck Biotechnology Resource Laboratory at Yale University, New Haven, CT).

S100A4Δ8C was mixed with the MIIA^1908-1923^ peptide in 1:1 ratio of S100A4 subunit per peptide and incubated for 1 hour at room temperature. Diffraction quality crystals of the complex were obtained by sitting drop vapor diffusion at 20°C by mixing 1 μl of the protein complex (15.5 mg/mL) with 1 μl of reservoir solution containing 0.1 M HEPES pH 7.0 and 30% (v/v) Jeffamine M-600. Rod-shaped crystals appeared after 3–4 days, which were flash cooled in liquid nitrogen without any additional cryo-protectant. Ca^2+^-bound S100A4Δ8C crystals were grown using a similar protocol except the reservoir solution contained 0.1 M HEPES pH 7.0, 0.1 M magnesium chloride, and 15% PEG 4000. Thin-plate shaped crystals appeared in one week and the crystals were harvested with mother liquor supplemented with 15% glycerol as a cryoprotectant for flash-cooling. Data were collected at beamlines X4A and X29A (Brookhaven National laboratory) for the S100A4Δ8C/MIIA^1908-1923^ peptide and Ca^2+^-bound S100A4Δ8C crystals, respectively. Data were integrated and scaled with HKL2000 [51]. The S100A4Δ8C/MIIA^1908-1923^ peptide complex crystals exhibited diffraction consistent with the space group P2_1_ (*a* = 30.28 Å, *b* = 91.99 Å, *c* = 32.86 Å, β = 112.6*°*) and apo-S100A4Δ8C crystals were triclinic with space group P1 (*a* = 28.80 Å, *b* = 34.36 Å, *c* = 95.31 Å, α = 95.48*°*, β = 95.30*°*, γ = 114.82*°*)*.*

### Structure determination

The S100A4Δ8C and S100A4Δ8C/MIIA^1908-1923^ peptide structures were determined by molecular replacement to 2.5 Å and 1.54 Å, respectively using the program MOLREP [52] and the Ca^2+^-S100A4 structure (PDB 2Q91) as the search model. Iterative cycles of model building and refinement were performed using COOT [53] and REFMAC5 [54]. For the S100A4Δ8C/MIIA^1908-1923^ peptide structure, continuous difference density was observed in the F_o_-F_c_ map contoured at the 3*σ* level following the first round of refinement, indicating the presence of a highly ordered peptide, asymmetrically bound to the S100A4 dimer. The final S100A4Δ8C/MIIA^1908-1923^ peptide model contained 2 S100A4 subunit chains (Ala2-Gly92) (named A and B), 1 myosin-IIA peptide, 4 calcium ions and 86 water molecules, resulting in a R_work_ and R_free_ of 20.9% and 25.1%, respectively (PDB 4ETO). The final Ca^2+^-S100A4Δ8C model contained 4 subunit chains (Cys3-Gly92), 8 calcium ions and 10 water molecules with a R_work_ and R_free_ of 22.8% and 27.7%, respectively (PDB 4HSZ).

### Anisotropy assays

Fluorescence anisotropy measurements were performed at 22°C using a Fluoromax-3-spectrofluorometer (Jobin Yvon). Individual reactions (200 μl) contained 50 nM FITC-MIIA^1904-1927^ and 0–30 μM S100A4Δ8C dimer in 20 mM Tris pH 7.5, 150 mM KCl, 1 mM DTT, 0.02% NaN_3_, 0.5 mM CaCl_2_ and 0.5 mg/ml BSA. For competition anisotropy measurements individual reactions contained 50 nM FITC-MIIA^1904-1927^, 0.3 μM S100A4 dimer and 0–10 μM MIIA^1851-1960^ dimer as described above. Controls included experiments representing maximum anisotropy (15 μM wild-type S100A4 and S100A4Δ8C, 50 nM FITC-MIIA^1904-1927^ and calcium) and minimum anisotropy (in the absence of S100A4 and in the presence of S100A4 and 5 mM EGTA). Anisotropy was measured using excitation and emission wavelengths of 490 nm and 520 nm, respectively. Measurements were acquired at the magic angle of 55° between the vectors of polarization of the excitation and emission light using a *G* factor of 0.634 as determined previously for FITC on this instrument. For measurements of S100A4Δ8C binding, data from two independent experiments were plotted using Graphpad Prism v5, and the dissociation constant was calculated by fitting to a single site saturation binding equation allowing for a floating Y_min_ value [11]. For competition anisotropy assays data from two independent experiments were plotted using Graphpad Prism v5, and fit to a sigmoidal dose–response equation with a variable slope to obtain the IC_50_ value.

### Promotion of disassembly assays

Assays comparing the ability of wild-type and the Δ8C S100A4 to depolymerize assembled myosin-IIA rods were performed as described in Li et al. [19], using 1.5 μM S100A4 dimer and 1.5 μM myosin-IIA rod dimer in 20 mM Tris–HCl pH 7.5, 150 mM NaCl, 1 mM DTT, 2 mM MgCl_2_, 0.5 mM CaCl_2_, and 0.02% NaN_3_. Reaction mixtures (100 μl) were incubated for 2 hr at 22°C and then centrifuged at 80,000 rpm (175000 *g*) for 10 min at 25°C in a TL-100 ultracentrifuge (Beckman). Samples of the reaction mixtures and supernatants were separated on a 12% Tris-Tricine SDS-polyacrylamide gel. Coomassie-stained gels were scanned and the extent of myosin-IIA polymerization was quantified by densitometry and analyzed with the ImageQuant version 5.2.

### NMR spectroscopy

The Ca^2+^-loaded S100A4Δ8C/MIIA^1908-1923^ and S100A4/MIIA^1893-1923^ samples were prepared as described previously [4] and contained 0.25 mM S100A4 subunit and 0.75 mM MIIA peptide in 10 mM CaCl_2_, 0.35 mM NaN_3_, 10 mM NaCl, 0.1 mM EDTA, 5 mM DTT, 10% D_2_O, and 10 mM Tris, and the pH was brought to 6.5 with HCl. The MIIA^1893-1923^-Cys-Proxyl peptide was titrated into the sample, and spectral changes were monitored after each addition via the ^15^N- HSQC signal. HSQC NMR data were collected at 37°C with a 600 MHz (600.13 MHz for protons) Bruker DMX NMR spectrometer equipped with pulsed-field gradients, four frequency channels, and a triple resonance, z-axis gradient cryogenic probe, Data were processed with NMRPipe [55], and proton chemical shifts were reported with respect to the H_2_O or HDO signal taken as 4.658 ppm relative to external trimethylsilylpropionic acid (0.0 ppm). The ^15^ N chemical shifts were indirectly referenced as described previously using the following ratio of the zero-point frequency: 0.10132905 for ^15^ N to ^1^H [56].

### Circular dichroism spectroscopy

MIIA^1851-1960^ was dialyzed into 20 mM Tris pH 7.5, 150 mM KCl, 0.5 mM CaCl_2_, 0.02% NaN_3_ and clarified by ultracentrifugation at 80,000 rpm for 10 minutes at 4°C in TLA 120.2 rotor. MIIA^1851-1960^ (40 μM monomer concentration) was transferred to a 1 mm path-length quartz cuvette and scanned in Jasco J-815 Spectrometer equipped with a PFD-425 temperature controller. Spectra from 190 to 260 nm were obtained at 22°C using a scan speed of 100 nm/minute, a 1 nm bandwidth, and a sensitivity of 100 mdeg. Thermal melt curves were obtained by monitoring ellipticity at 222 nm while the sample temperature was increased from 2 – 85°C in 2°C intervals at a rate of 60°C/h, using a 1 nm bandwidth. The data were fit to a sigmoidal dose–response with a variable slope using Graphpad Prism v5.

### Analytical ultracentrifugation

Sedimentation equilibrium and velocity experiments were conducted at 22°C in a Beckman XL-I analytical ultracentrifuge using a Ti60 rotor and Raleigh interference optics. Wild-type S100A4 and MIIA^1851-1960^ were dialyzed into 20 mM Tris pH 7.5, 150 mM KCl, 0.5 mM CaCl_2_, 1 mM TCEP and 0.02% NaN_3_ and clarified by ultracentrifugation at 80,000 rpm for 10 minutes at 4°C in TLA 120.2 rotor. Sedimentation equilibrium analysis of MIIA^1851-1960^ and S100A4 was conducted in six channel centerpieces by sequentially equilibrating the protein at three concentrations for 24 hr at 12,000, 20,000 and 28,000 rpm and globally analyzing the data using HeteroAnalysis v1.1.44 (James L. Cole and Jeffrey W. Lary). The sedimentation velocity experiments were conducted at 53,000 rpm using double sector centerpieces and analyzed using DCDT + v2.4.0 [57,58]. The best-fit parameters and their 95% joint confidence intervals are reported. The calculated masses of the wild-type S100A4 and MIIA^1851-1960^ monomers are 11,597.3 and 12,397.5 Da, respectively. Values of ν-bar = 0.715 for S100A4/MIIA^851-1960^ and 0.735 for S100A4, ρ = 1.0059, μ = 1.00586 cp were calculated using Sednterp v1.06 (Hayes, B., T. Laue & J. Philo, Sedimentation Interpretation Program. 2003, University of New Hampshire) from the amino acid and buffer compositions, respectively.

## Abbreviations

MIIA: Non-muscle myosin-IIA; EF: EF-hand; FITC: Fluorescein isothiocyanate; DTT: Dithiothreitol; TCEP: Tris (2-carboxyethyl) phosphine.

## Competing interests

The authors declare that they have no competing interests.

## Authors’ contributions

UAR carried out the X-ray crystallography studies. NGD purified all the proteins and performed all biochemical analyses. KMV and SN performed the NMR studies and PTW prepared the spin-labeled peptides. MB performed and analyzed the analytical ultracentrifugation data. DJW, SCA and ARB designed the study, analyzed the data and wrote the manuscript. All authors read and approved the final manuscript.

## Supplementary Material

Additional file 1: Table S1 Summary of Sedimentation Velocity Data. **Figure S1:** Representative scans and residuals for sedimentation equilibrium measurements of MIIA^1851-1960^. The solid lines represent the best fit from a global nonlinear least-squares analysis of data obtained at 20,000 and 28,000 rpm. (A) 0.5 mg/ml, (B) 1.0 mg/ml and (C) 2.5 mg/ml MIIA^1851-1960^ (40.3, 80.6 and 201.7 μM monomer concentration). **Figure S2**: Sedimentation velocity of the S100A4/MIIA^1851-1960^ complex. Plots of sedimentation coefficient distribution g*(s) versus S_20,w_ for S100A4 alone (A) and MIIA^1851-1960^/S100A4 mixtures at a molar ratio of 0.25:1, MIIA^1851-1960^ dimer:S100A4 dimer. The green line is the best fit to the S100A4 dimer, the blue line is the best fit to the S100A4 dimer/MIIA^1851-1960^ monomer complex and the red line is the best fit for all species in the S100A4/MIIA^1851-1960^ mixtures. **Figure S3**: Interaction of S100A4Δ8 molecules in the crystal lattice. Click here for file
