# Supplementary material for: Structure of the S100A4/myosin-IIA complex
Source: BMC Struct Biol. 2013 Nov 20;13:31. doi: 10.1186/1472-6807-13-31 (PMC3924328; doi:10.1186/1472-6807-13-31)
Supplement: Additional file 1: Table S1 — Summary of Sedimentation Velocity Data. Figure S1: Representative scans and residuals for sedimentation equilibrium measurements of MIIA1851-1960. The solid lines represent the best fit from a global nonlinear least-squares analysis of data obtained at 20,000 and 28,000 rpm. (A) 0.5 mg/ml, (B) 1.0 mg/ml and (C) 2.5 mg/ml MIIA1851-1960 (40.3, 80.6 and 201.7 μM monomer concentration). Figure S2: Sedimentation velocity of the S100A4/MIIA1851-1960 complex. Plots of sedimentation coefficient distribution g*(s) versus S20,w for S100A4 alone (A) and MIIA1851-1960/S100A4 mixtures at a molar ratio of 0.25:1, MIIA1851-1960 dimer:S100A4 dimer. The green line is the best fit to the S100A4 dimer, the blue line is the best fit to the S100A4 dimer/MIIA1851-1960 monomer complex and the red line is the best fit for all species in the S100A4/MIIA1851-1960 mixtures. Figure S3: Interaction of S100A4Δ8 molecules in the crystal lattice. [file 1472-6807-13-31-S1.doc]

Additional file 1. Table S1: Summary of Sedimentation Velocity Data

| MIIA^1851-1960^: S100A4 | [Peak 1] ^a^  (green) | [Peak 2] ^a^  (blue) | Peak 1  S_20,w_ | Peak 1  MW (kDa) | Peak 2  S_20,w_ | Peak 2  MW (kDa) |
| --- | --- | --- | --- | --- | --- | --- |
| MIIA^1851-1960^ | 2.09 ± 0.02 | – | 1.91 ± 0.01 | 18.5 ± 0.2 | – | –- |
| 0.5 : 1.0 | – | 2.36 ± 0.01 | – | – | 2.87 ± 0.01 | 31.1 ± 0.2 |
| 1.0 : 1.0 | 1.0 ± 0.1 | 2.50 ± 0.01 | [1.9] | [19.0] | 2.89 ± 0.01 | 31.5 ± 0.2 |
| 1.5 : 1.0 | 1.8 ± 0.2 | 2.4 ± 0.3 | [1.9] | [19.0] | 2.90 ± 0.06 | 31.6 ± 3.7 |
| S100A4 | 2.00 ± 0.02 | – | 1.90 ± 0.01 | 21.2 ± 0.3 | – | – |
| 0.25 : 1.0 | 1.1 ± 0.1 | 2.4 ± 0.1 | [1.9] | [21.0] | 2.91 ± 0.02 | 31.5 ± 0.6 |

^a^Relative amount of plateau concentrations.

Values in square brackets are fixed.

Errors represent the average joint confidence interval for 1 standard deviation.


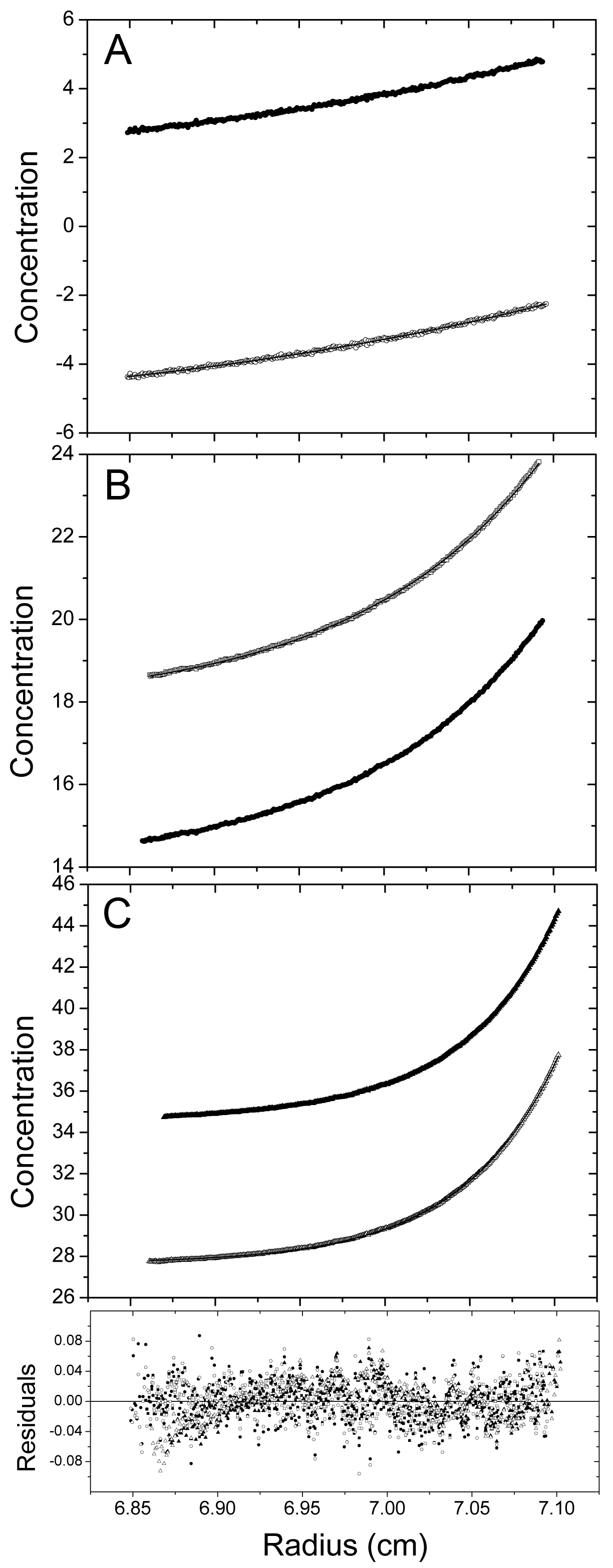


Figure S1. Representative scans and residuals for sedimentation equilibrium measurements of MIIA^1851-1960^. The solid lines represent the best fit from a global nonlinear least-squares analysis of data obtained at 20,000 and 28,000 rpm. (A) 0.5 mg/ml, (B) 1.0 mg/ml and (C) 2.5 mg/ml MIIA^1851-1960^ (40.3, 80.6 and 201.7 µM monomer concentration).


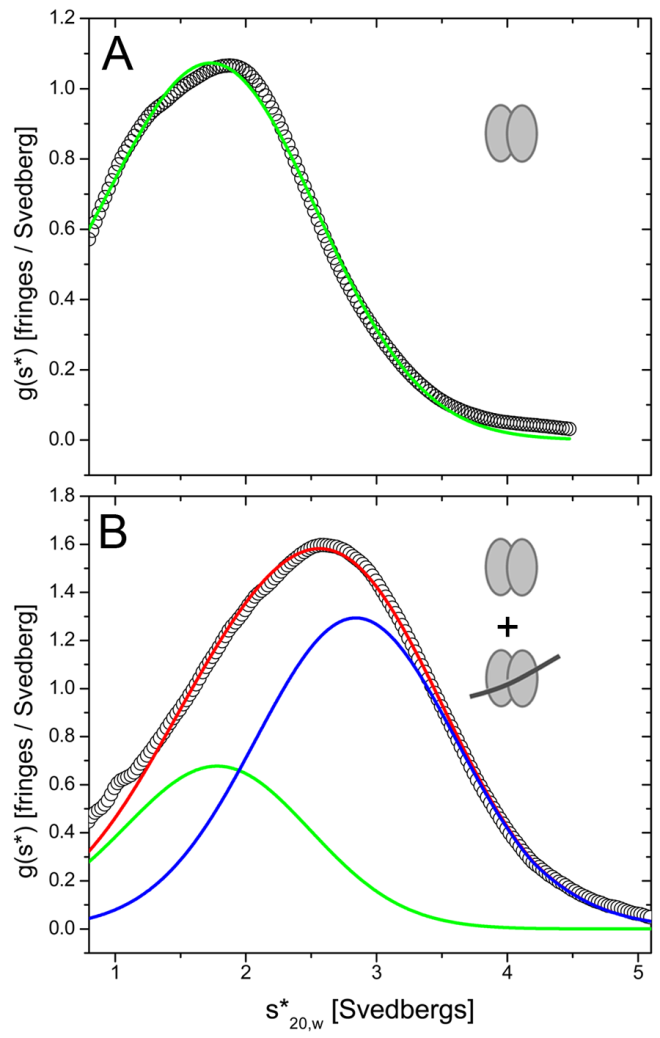


Figure S2. Sedimentation velocity of the S100A4/MIIA^1851-1960^ complex. Plots of sedimentation coefficient distribution g*(s) versus S_20,w_ for S100A4 alone (A) and MIIA^1851-1960^/S100A4 mixtures at a molar ratio of 0.25:1, MIIA^1851-1960^ dimer:S100A4 dimer. The green line is the best fit to the S100A4 dimer, the blue line is the best fit to the S100A4 dimer/MIIA^1851-1960^ monomer complex and the red line is the best fit for all species in the S100A4/MIIA^1851-1960^ mixtures.


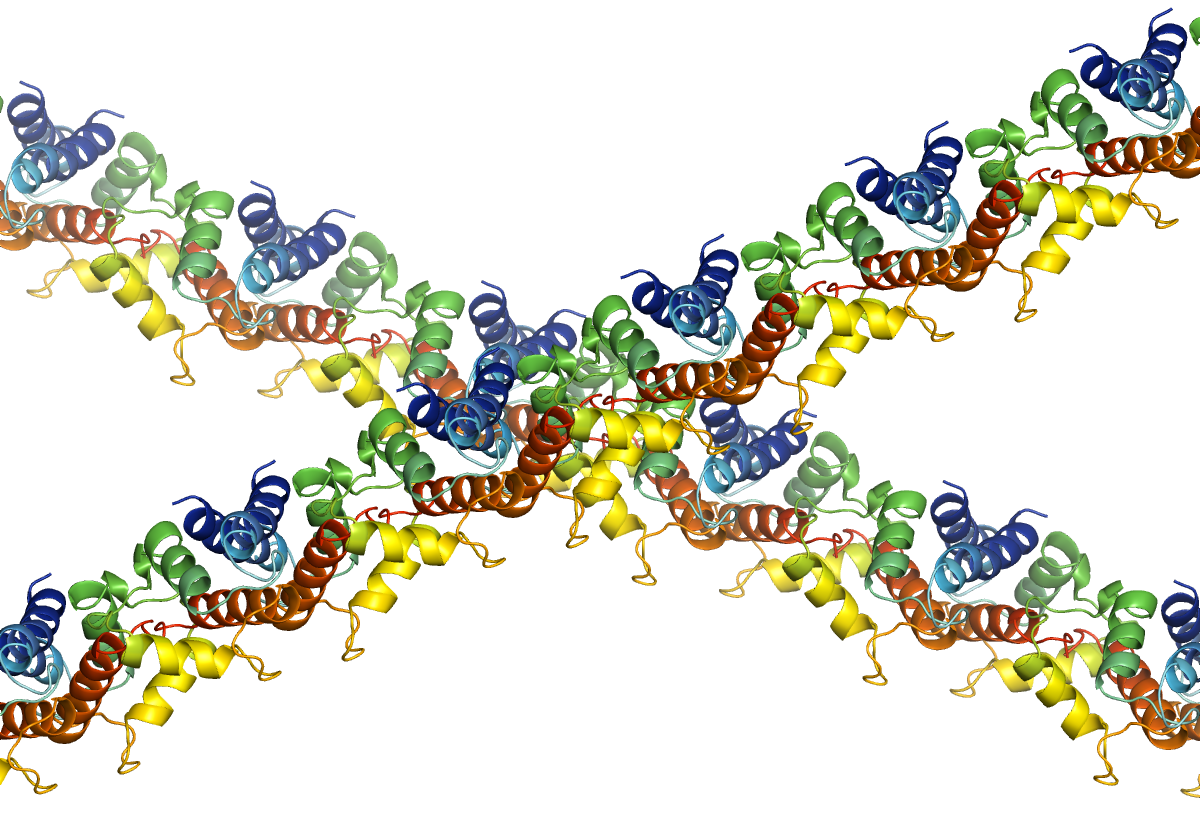


Figure S3. Interaction of S100A4Δ8 molecules in the crystal lattice.
